# Adopting Machine Learning and Spatial Analysis Techniques for Driver Risk Assessment: Insights from a Case Study

**DOI:** 10.3390/ijerph17145193

**Published:** 2020-07-18

**Authors:** Muhammad Zahid, Yangzhou Chen, Arshad Jamal, Khalaf A. Al-Ofi, Hassan M. Al-Ahmadi

**Affiliations:** 1 College of Metropolitan Transportation, Beijing University of Technology, Beijing 100124, China; zahid@emails.bjut.edu.cn; 2 College of Artificial Intelligence and Automation, Beijing University of Technology, Beijing 100124, China; 3 Department of Civil and Environmental Engineering, King Fahd University of Petroleum & Minerals KFUPM BOX 5055, Dhahran 31261, Saudi Arabia; arshad.jamal@kfupm.edu.sa (A.J.); kaluwfi@kfupm.edu.sa (K.A.A.-O.); ahmadi@kfupm.edu.sa (H.M.A.-A.)

**Keywords:** aggressive driving, traffic violations, inverse distance weighted (IDW) interpolation, geographic information system (GIS), machine learning

## Abstract

Traffic violations usually caused by aggressive driving behavior are often seen as a primary contributor to traffic crashes. Violations are either caused by an unintentional or deliberate act of drivers that jeopardize the lives of fellow drivers, pedestrians, and property. This study is aimed to investigate different traffic violations (overspeeding, wrong-way driving, illegal parking, non-compliance traffic control devices, etc.) using spatial analysis and different machine learning methods. Georeferenced violation data along two expressways (S308 and S219) for the year 2016 was obtained from the traffic police department, in the city of Luzhou, China. Detailed descriptive analysis of the data showed that wrong-way driving was the most common violation type observed. Inverse Distance Weighted (IDW) interpolation in the ArcMap Geographic Information System (GIS) was used to develop violation hotspots zones to guide on efficient use of limited resources during the treatment of high-risk sites. Lastly, a systematic Machine Learning (ML) framework, such as K Nearest Neighbors (KNN) models (using k = 3, 5, 7, 10, and 12), support vector machine (SVM), and CN2 Rule Inducer, was utilized for classification and prediction of each violation type as a function of several explanatory variables. The predictive performance of proposed ML models was examined using different evaluation metrics, such as Area Under the Curve (AUC), F-score, precision, recall, specificity, and run time. The results also showed that the KNN model with k = 7 using manhattan evaluation had an accuracy of 99% and outperformed the SVM and CN2 Rule Inducer. The outcome of this study could provide the practitioners and decision-makers with essential insights for appropriate engineering and traffic control measures to improve the safety of road-users.

## 1. Introduction

Road transport is considered the backbone of the nation’s economy. In China, rapid economic growth during the past three decades has brought a revolution in the transportation industry. The motorization rate has witnessed exponential growth, particularly in urban areas. Though this rapid expansion of urban transport infrastructure has inarguable benefits for various businesses, it has caused serious agony in the form of extreme traffic congestion, limited parking facilities, increases air pollution, and noise pollution, as well as safety concerns. For example, a study reported that a total of 244,937 road accidents occurred in China in 2018, resulting in 63,194 deaths, 258,532 injuries, and an overall direct economic loss of 1.38 billion yuan [1]. Road safety has become a global challenge in the era of rapid motorization. Traffic crashes severely affect public health, and further pose huge socio-economic losses every year. It is estimated that around 1.35 million people are killed, and over 50 million others sustain injuries due to traffic crashes every year, causing over $520 billion losses to the global economy [2]. It is essential to explore the underlying factors and to identify high-risk zones to mitigate the burden of such unfortunate events. Numerous studies have examined the factors contributing to crash occurrences and their severity outcome; however, crashes are random events having spatio-temporal variations that warrant comprehensive investigation under given circumstances [3,4]. Several previous studies suggest that driver-related factors (mainly including distractions, fatigue driving, drunk driving, non-compliance to traffic rules) are responsible for over 90% of total crashes [5,6]. Among all driver attributes, only a few studies have focused on analyzing drivers’ violation patterns usually caused by aggressive driving behavior [7,8]. Alonso et al. conducted a questionnaire-based study to investigate the tendencies and perceptions of a sample of Spanish drivers (*n* = 1100) toward traffic norms [9]. It was concluded that the vast majority of respondents believed that the established norms were effective in improving road safety.

Association of aggressive driving behavior and traffic violation with crash characteristics has been the subject of burning research in recent years. Studies suggest that several factors are responsible for traffic violations inspired by aggressive driving behavior. The key in this regard may be grouped into four categories: psychological (aggressive nature, anxiety, stress, hatred, competition, gender), social (the presence of passengers in the vehicle; the gender and age of the individuals demonstrated aggressiveness), temporal (time pressure and daytime), and environment-related factors (road conditions, traffic density, and weather) [10,11]. A series of research investigated the association between crashes and traffic violations. Studies reported that drivers involved in deadly crashes were found to have more charges of traffic violations than non-guilty drivers [12,13]. Similarly, previous research also showed that drivers who were previously involved in frequent violations were at high risk of involving subsequent crash [14,15]. It is acknowledged that inexperienced drivers (particularly young drivers) do not have adequate driving skills, yet most of them overestimate their driving abilities and therefore do not consider the various hazards as risky while driving [16]. Studies showed that male drivers are usually more likely to commit traffic violations and consequently have a higher chance of involvement in serious/fatal traffic accidents [17,18,19,20,21]. A recent study concluded that the elderly age is less indicative of traffic violations compared to the young driving population [22]. It is established that strict enforcement could discourage the drivers from committing traffic violations, and significantly reduce the number of crashes, as well as their severity [16]. Similarly, studies have shown that pro-active traffic control and forecasting could be very beneficial to monitor dynamic drivers maneuvers, thus ensuring strict compliance traffic regulations and mitigate congestion in urban areas [23,24,25].

Researchers have utilized different analysis techniques for characterization and detailed prognosis of traffic violations. For example, Firth’s penalized logistic regression, logistic regression, and generalized order logit models have been widely used to investigate wrong-way driving crash information in urban areas [26,27,28]. Lucidi et al. assessed the validity of the Ulleberg and Rundmo model to predict risky driving behaviors (considering violations, lapses to response, and errors) among large samples of older drivers population [29]. In another study, researchers evaluated 11,055 cases for overspeeding reported during the period 2006–2010 in Guangdong Province, China [30]. It was found that private cars, lack of adequate street lighting at night, and low visibility were the critical factors associated with the overspeeding violations. Studies have also focused on investigating the factors contributing to overspeeding violations for individual vehicle types, such as cars/taxis [31] and trucks [32]. The findings indicated that age, employment, mental health, and driving status were significantly associated with overspeeding truck violations. For taxis, drivers’ age, the work experience, the driving style, and daily driven kilometers were all linked to the overspeeding profiles.

Tselentis et al. utilized Data Envelopment Analysis (DEA) based framework for evaluation and benchmarking of drivers’ safety efficiency under a naturalistic driving environment [33]. The driver population (N = 56) was divided into three categories, i.e., less efficient, weakly efficient, and most efficient. Eboli et al. in their study, proposed a novel framework considering kinematic parameters, such as speed, lateral, and longitudinal acceleration profiles to establish whether driver behavior was safe or unsafe [34]. Jovanovic et al. investigated the speeding violation factors and assessed the predictive validity of the adjusted Theory of Planned Behavior (TPB) model in association with speed violation [35]. Warner and Aberg attempted to explore about drivers’ perspective of speeding violation and predicted the speeding intent. A total of 162 car owners were selected for the analysis. The findings revealed that the indicators, including mood, attitude, social norms, and perceived behaviors, seem to be more effective in predicting the speeding intentions of the drivers [36]. Likewise, Kim et al. introduced a method for regulating parking violations using camera captured images through computer vision techniques. The result showed that the illegal parking was determined by the conformity of the lanes and the vehicle’s shadow [37]. De Winter and Dodou conducted a detailed meta-analysis that revealed that age and annual mileage have a great deal of association with drivers’ errors and violations. The authors found that young driving age appeared to be associated positively with violations and errors. Toledo et al. assessed the potential for an in-vehicle recorder system to monitor road-driver behavior [38]. Implementation of the proposed system using the Drive Diagnostics system showed that short-term rates and risk indices might be reduced. In another study, researchers designed a combined method considering both objective and subjective parameters to identify crash risk levels [39]. Based on the study results, the authors classified three ranges for being involved in a crash, i.e., low, medium, and high.

In recent years, major Chinese metropolitans witnessed an increasing trend in traffic violations. These violations account for 75% of total crashing occurring in the country [40]. Traditionally, statistical modeling or simulation-based approaches have been widely used for examining aggressive driving behavior and analysis of traffic violations. However, these methods have several underlying assumptions and are unable to estimate associations between predictor variables in a realistic fashion. Further, a vast majority of such studies have focused on analyzing the patterns of traffic violations in the urban metropolitan, whereas factors contributing to traffic violations along expressways have been scarcely explored. To fill this gap, we utilized state-of-the-art Machine Learning (ML) models to predict violations taking into account various spatio-temporal attributes. The main contributions of current work are: (i) we advocate the application of Inverse Distance Weighted (IDW) method of interpolation in ArcMap (Geographic Information System (GIS)) to identify violation hotspots along expressways; (ii) proposed a systematic ML framework, including SVM, CN2 Rule Inducer, and K Nearest Neighbors (KNN) to classify and predict traffic violations considering a number of available explanatory variables; (iii) performed comprehensive comparative analysis for proposed ML algorithms based on several classification evaluation metrics; and (iv) our results showed that KNN (with k = 7) outperformed other models.

The remainder of the paper is structured as follows. Section 2 presents description of study area, data collection, and detailed methods for hotspot analysis and violation prediction using ML. Section 3 provides study results and discussion, highlighting key descriptive anlaysis, mapping of violation hotspots, ML models’ prediction comparions, and Spearman correlation analysis. Finally, Section 4, provides conclusions, study limitations, and outlook for future studies.

## 2. Data and Methods

### 2.1. Selection of Study Area

**Figure 1:**
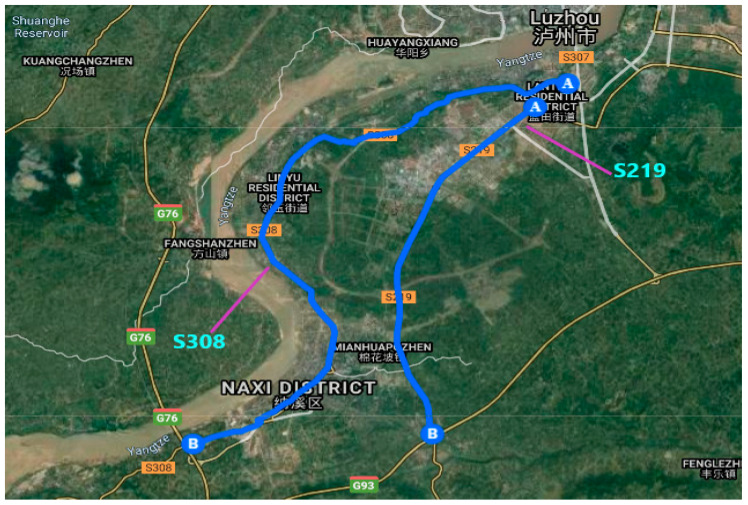
Locations of S219 and S308 in the study area (from Google Maps).

The city of Luzhou (shown in Figure 1) was selected as the study area. It is a prefecture-level municipality with an area of 12,246 km^2^ and a population over 1 million and is located in the southeast of Sichuan Province, China. Located at the combination of the Tuo River and Yangtze River, the Luzhou port on the Yangtze River is the major port of Sichuan since the Chongqing Province in 1997 [41]. As per the National Bureau of the Statistics People’s Republic of China (PRC), by 2017, the country had 4.77 million of paved roads and over 300 million registered vehicles [1].

### 2.2. Data Collection

Traffic violation data for the year 2016 was obtained from the department of Sichuan traffic police in Luzhou city and collected from off-site traffic enforcement cameras system. Therefore, traffic violations along expressways road segments may be missing. It is worth stating that the license plate number of vehicles was not available in the dataset, which bounds its application to record a particular driver’s history of the violation. It is a significant shortcoming that could be addressed in future research for better analysis of violations by various vehicle types’ drivers.

### 2.3. GIS-Based Analysis for Violation Hotspots

Spatial Statistic toolbox in ArcMap was used for distribution and identification hotspots along the two expressways. Four steps were carried out to find the hotspots in the study area. In the first step, traffic violation point data was loaded in GIS. Each point represented a traffic violation caused by any of the four types, i.e., Violation of prohibited markings, Wrong-way driving, Illegal parking, or Overspeeding. In the second step, Data clusters were created in order to convert the data to a weighted point. A spatial statistics tool (Collect Event) was used in ArcMap in order to convert the data to a weighted point. Weights were assigned based on the frequency of the traffic violation at a particular point. Collect Event tools combines all coincident points that have the same X and Y centroid coordinates. During the third step, the Getis-Ord statistic was used to identify traffic violation hotspots. A high value of Getis-Ord statistic shows that a cluster is having high index values (hot spots), and a low value of Getis-Ord statistic represents a cluster of low index values (cold spots). Mathematically, the Getis-Ord statistic and its z-score are expressed by the following equations.

(1) $$G_{i}^{*}(d)=\frac{\sum_{j=1}^{n}w_{ij}(d)x_{j}}{\sum_{j=1}^{n}x_{j}},$$

(2) $$Z(G_{i}^{*})=\frac{G_{i}^{*}-E(G_{i}^{*})}{\sqrt{VAR(G_{i}^{*})}},$$

where $G_{i}^{*}$ represent spatial dependency of the incident i, $x_{j}$ is feature value for j, $w_{ij}$ is the spatial weights for i, and j stands for distance d. n is the total number of features.

Inverse Distance Weighted (IDW) method of interpolation was used on these hotspots to estimates traffic violations along the expressways. IDW helps in estimating the neighboring values by averaging the values of sample data points. The principle of IDW is, the closer a point is to the center of the cell being estimated, the more influence or weight it has in the averaging process.

### 2.4. Traffic Violation Prediction Using ML 

Three different ML algorithms, including K Nearest Neighbors (KNN), Support Vector Machine (SVM), and CN2 Rule Inducer, were implemented for predicting traffic violations using the available violation data collected from the study area. The three algorithms were implemented using the orange data mining toolbox in python. The preliminaries and detailed methodology of proposed ML methods are discussed in the following passages.

#### 2.4.1. K Nearest Neighbors (KNN)

The K-nearest neighbor (KNN) classifier is a conventional non-parametric classifier initially proposed by Cover and Hart in 1967 [42]. It is a low computation complexity method for object recognition and classification tasks, such as character, face, and other objects. The KNN principle is based on an intuitive idea that the data points of the same class should be nearer to the feature space. The very first step of implementation is the collection of traffic violations observations and is classified $C=\{C_{1},C_{2},\ldots ,C_{N}\}$. Firstly, KNN determines the distances for each training sample and the target point, then chooses the closest k-samples to the target. Such k-samples assess together the target point class. The distance measurement of the attributes is a simple way of expressing the point’s resemblance. Afterward, the shortest k distance $D=\{d_{1},d_{2},d_{3},\ldots \ldots ,d_{k}\}$ is chosen where each neighbor belongs among N classes to a specific class $Y=\{y_{1},y_{2},\ldots \ldots ,y_{n}\}$. Given a data set labeled with observations $\left(x_{i},y_{i}\right)$ and i=1, 2,…,n to capture the relationship between x data and y label. More explicitly, to know a function g : X→Y such that g (x) can accurately predict the corresponding output class Y given an unknown observation X. Moreover, the distance between the components to be recognized, and each class is then computed by euclidean, manhattan, mahalanobis and weighted by uniform and distance techniques. The distance can be defined as the nearer query neighbors point have a more significant impact than neighbors further away, while uniform described all points in each neighborhood are weighted equally. The formula for euclidean, manhattan, and mahalanobis are given in below equations,

(3) $$D(x,y)=\sqrt{\sum_{i=1}^{n}\left(x_{i},y_{i}\right)},$$

(4) $$D(x,y)=\sum_{i=1}^{n}\left|x_{i}-y_{i}\right|,$$

(5) $$D(\vec{x},\vec{y})=\sqrt{{\left({\vec{x}}_{i}-{\vec{y}}_{i}\right)}^{T}S^{-1}({\vec{x}}_{i}-{\vec{y}}_{i})},$$

where D denotes distance, and $S^{-1}$ is the covariance of the matrix of ${\vec{x}}_{i}$ and ${\vec{y}}_{i}$. The weight distribution is achieved through distance and uniform techniques, and the k values assigned for these different metrics are given in Table 1.

#### 2.4.2. Support Vector Machine (SVM)

The Support Vector Machine (SVM) method is a supervised learning method proposed by Boser et al. in 1992 [43]. It seeks to find the ideal hyperplane, which divides two or more classes by seeking the maximal margin distances (e.g., positive vs. negative). In the classification scenario, the SVM seeks for the curve that can separate and classify the training data, ensuring that the difference between the curve, as well as other training class observations (support vectors), is as high as possible. This separation can be achieved in the same space or an ample space by mapping the input space into a feature space through a kernel function (radial basis function (RBF), polynomial, sigmoid, etc.). The training dataset of n points with observations $x_{i}$ i=1, 2,…,n is defined as the vectors relative to the class observations $y_{i}$ i=1, 2,…,n. In particular, SVM adjusts the balance between the margin as well as the error by adjusting a C parameter. With a higher optimum performance due to less computational difficulties and reasonable precision that reduced overfitting, the RBF kernels have been selected for the ultimate model of our research. The SVM model was implemented using the Orange python scripting tool. The formula for the RBF kernel is given below.

(6) $$K(x,y)=\exp(-\gamma {\left|x_{i}-y_{i}\right|}^{2}),\;\gamma \;>\;0$$

where K represents kernel, and γ can be termed kernel ‘spread’, as well as the decision region. The values of γ and C are 10 and 1.

#### 2.4.3. CN2 Rule Inducer

The rule learning model for traffic violations and classification were discussed in this study. The CN2 Rule Inducer is a classification method designed to generate simple output, if condition then forecasts class, even in conditions where noise can occur. Moreover, CN2 Rule Inducer generates a class distribution based on the number of instances covered and distributed over the classes. In other words, it indicates the total number of representatives of the class. In our study, we employed a statistical significance check to determine whether the new rule has a valid correlation between features and classes. In addition, rules are pre-determined using two methods: (i) statistical likelihood ratio (SLR or LRS) tests and (ii) minimum threshold for rules coverage. The LRS test further shows two tests: firstly, the minimum level of relevance of a rule α_1_, and the second LRS test is equivalent to its parent rule, since it examines whether the last classification of rule is of adequate significance α_2_. In our implementation, we introduced exclusive coverage at the upper stage, such as an unordered rule, while Laplace estimation was used at the lower level for function evaluation. Laplace estimation has described as an alternative measure of the quality of the rule to correct undesirable entropy (downward bias) as follows:

(7) $$Laplace\;Estimation\;(R)=\frac{p+1}{p+n+k},$$

where ‘R’ is the rule, ‘p’ refers to the number of positive examples defined in the training set covered by the rule ‘R’, ‘n’ refers to the number of negative examples by ‘R’, and ‘k’ is the number of classes included in the training set. The values used for LRS tests are shown in Table 2, whereas the CN2 Rule Inducer viewer listed in Table A1 in Appendix A was obtained using stratified 10-fold cross-validation.

### 2.5. Performance Evaluation Metrics for ML Models

Different classification evaluation metrics were used to assess and compare the predictive performance of proposed ML methods. These include; precision, recall, F-score, accuracy, and specificity. Precision quantifies the number of positive predictions that are made correctly, while the recall quantifies the number of correct positive predictions that could have been made from all the positive predictions. The formula for calculating precision and recall could be found in Equations (8) and (9). The F-score comprises both the recall and the precision and is calculated from Equation (10). Accuracy is the proportion of the correct sample to the total number of samples and can be calculated from Equation (11). Similarly, specificity can be calculated from Equation (12).

(8) $$Precision=\frac{TP}{TP+FP},$$

(9) $$Recall=\frac{TP}{TP+FN},$$

(10) $$F-score=\frac{1}{Precision}+\frac{1}{Recall},$$

(11) $$Accuracy=\frac{TP+TN}{TP+TN+FP+FN},$$

(12) $$Specificity=\frac{TN}{TN+FP}.$$

## 3. Results and Discussions 

### 3.1. Analysis of Descriptive Statistics 

Traffic violations experienced by different vehicles, like private cars, taxis, vans, buses, and small trucks, were included in this analysis since they hold a large proportion of total occurrences of violations. A total number of 2003 violations by different vehicles were used for the analysis once the data was pre-processed. The occurren`ce of violations, comprised of time, date, month, day of the week, and time of the day, was assessed. The location of the violation was just to approximate the position of the occurrence of a traffic violation. In the present work, this detail was used to consider the spatial distribution of various type of violations occurred on urban expressways. Key descriptive statistics for data used in this research are summarized in Table 3.

Violations committed by taxi drivers and private cars were more prevalent compared to other vehicles. Wrong-way driving (74.94%) comprised the highest proportion of observed violation followed by violation of prohibited road marking (17.87%), overspeeding (4.54%), and illegal parking (2.65%). The main reason for a significantly high percentage of wrong-way driving violations may be attributed to the fact that vehicles (taxis and private cars drivers in particular) tend to use the wrong way to avoid long travel to the next entrance or exit ramp to save time. In comparison, a relatively low percentage of overspeeding violations may be attributed to the presence of speed surveillance cameras at multiple locations along both expressways. Since wrong-way driving usually results in more severe crashes due to head-on collisions, it is essential to identify high-risk areas and factors that are likely to encourage wrong-way driving. Considering the distribution of violations caused by different vehicle types, it may be noted from Table 3 that private cars were involved in approximately three-fifths (58%) of the total violations, while violations caused by buses accounted for only 2.55% of total violations. Considering the temporal distribution of violations, the Spring season had the highest percentage of reported violations (54.72%), followed by winter (34.40%), whereas Autumn had the lowest proportion (3.10%) of total violations. The large proportion of Spring violation may be associated with frequent travel during this season. Similarly, weekdays and peak periods accounted for almost 70% and 48% of reported violations, respectively.

### 3.2. Mapping of Violation Hotspots 

**Figure 2:**
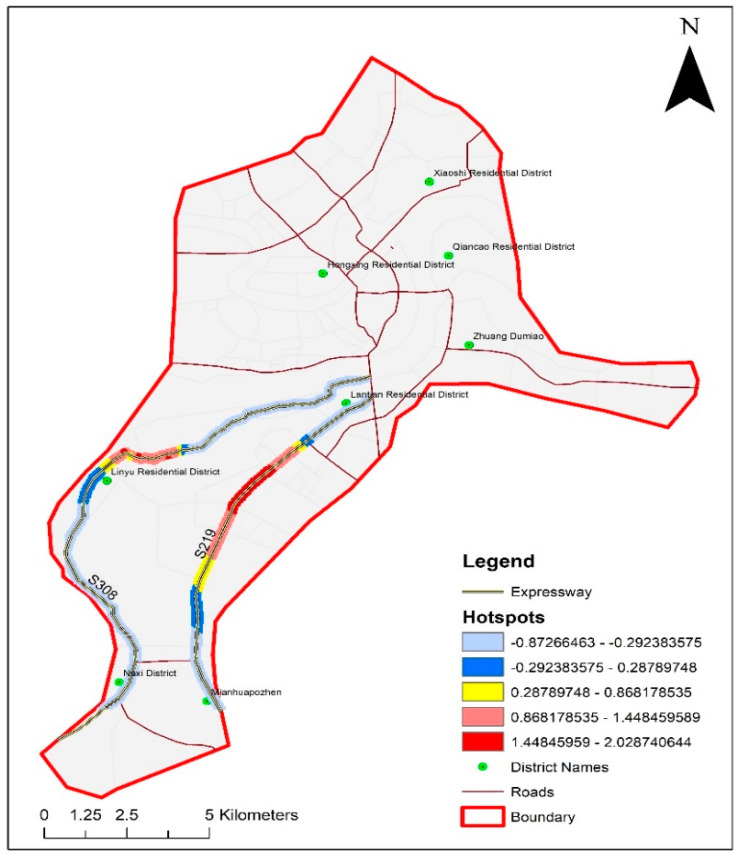
Violation hotspots mapping in the study area

Figure 2 shows the mapping of violations based on frequency-based clustering and the IDW method in ArcMap GIS. A total of 2003 traffic violations were observed along two expressways S219 and S308. S219 connects two residential districts, Lantian Residential District and Mianhuapozhen, in Luzhou, Sichuan, China. This expressway passes through various residential zones, one commercial zone, and one public facilities zone. S308 connects Lantian Residential District, Linyu Residential District, and Naxi District in the study area. As shown in Figure 2, both expressways had one major hotspot. The hotspot is located along the commercial and public facility zones. Wrong-way driving was the most observed traffic violation along both expressways. This observation may be attributed to the fact that drivers usually tend to drive the Wrong-way to avoid long travel along the expressway to reach their destinations or nearby residential areas to maximize profit. Expressway S219 had more number of hotspots compared to S308 due to densely populated residential zones on both sides in the vicinity of observed hotpots. Instead, S308 is occupied by coldspots, as shown in Figure 2. This observation is intuitive because this expressway runs along the rivers, and it does not divide any residential areas. Secondly, there are no commercial or public facilities along this expressway. Hence, very few traffic violations are observed along S308. The hotspot along this road is near to the airport along the curve (shown in Figure 2). More number of violations in these areas could be attributed to the presence of airport and sharp curve along the expressway. In general, hotspots frequency analysis along both expressways was dominated by wrong-way driving, followed by overspeeding, illegal parking, and violation of prohibited road markings.

### 3.3. ML Model’s Comparison for Violation Prediction

**Figure 3:**
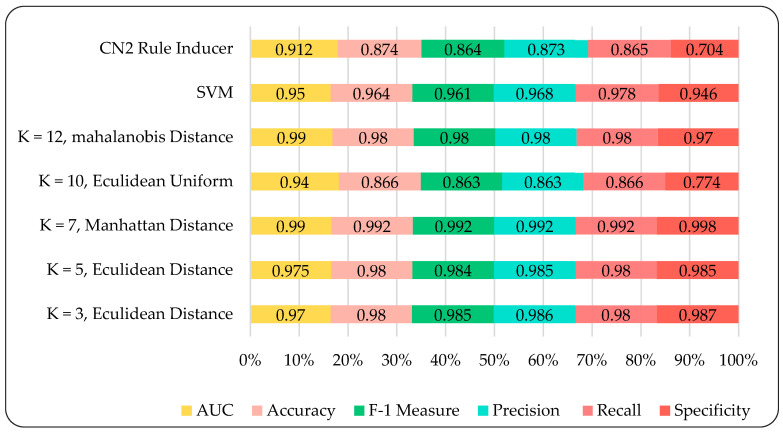
Performance comparison of different models.

**Figure 4:**
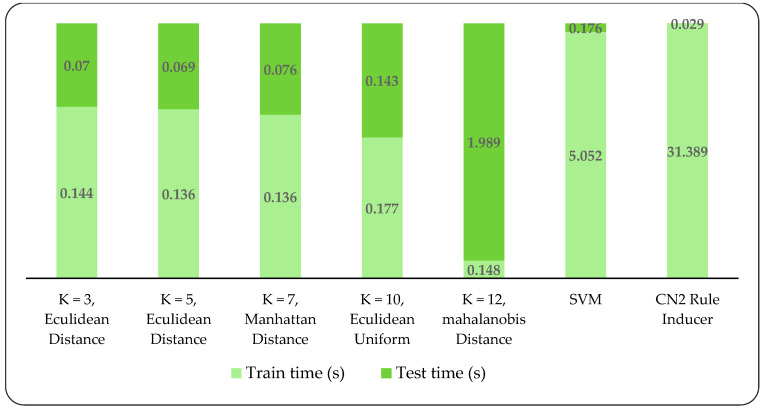
Run time (s) comparison of different models.

A detailed comparative analysis was conducted to examine the applicability and efficacy of applied models. The model’s performance is checked in terms of area Under the curve (AUC), accuracy, precision, recall, log loss, specificity, and F-1 score. Amongst these models, the KNN model outperformed the CN2 Rule Inducer and SVM model. As shown in Figure 3, we considered KNN with different k neighbors 3, 5, 7, 10, and 12 of different metrics and weights. The accuracy achieved for these different k neighbors is 99, 98, 98, 87, and 98 percent, respectively. Besides, all these k neighbors achieved higher accuracy and performed better except k = 10 nearest neighbor of uniform euclidean with obtained accuracy 87 percent. Hence, the average KNN model accuracy is 97.3 percent, which indicates that the predictive performance of KNN is more robust compared to SVM and CN2 Rule Inducer. Figure 3 also shows the obtained AUC, accuracy, precision, recall, F-1 score, and specificity for SVM are 0.95, 0.964, 0.963, 0.978, 0.961, and 0.945. Similarly, the AUC, accuracy, precision, recall, F-1 score, and specificity for CN2 Rule Inducer are 0.91, 0.874, 0.873, 0.865, 0.864, and 0.70. In contrast, the KNN model takes less training time and test time with different KNN (k = 1, 3, 5, 7, 10, and 12). Additionally, the KNN model takes less training time than the SVM and CN2 rule inducer, which therefore validates the efficiency of the KNN model. The models’ run times are shown in Figure 4.

### 3.4. Spearman Correlation Analysis

**Figure 5:**
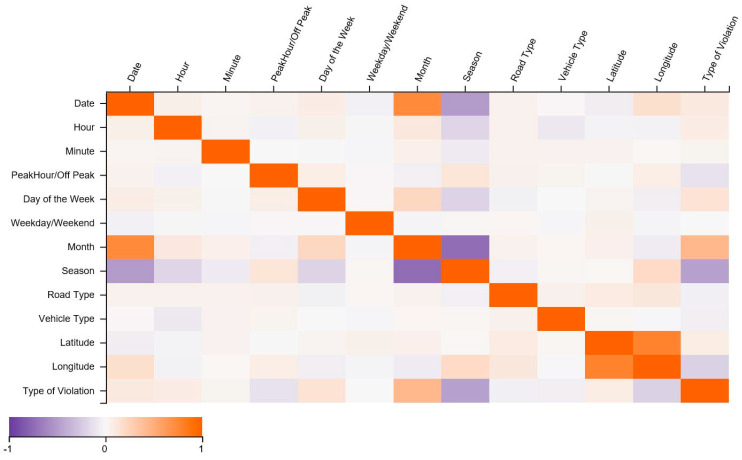
Variables correlation matrix.

After data pre-processing, we estimate the rank correlation coefficient of the Spearman between the two features and obtain the matrix of the correlation coefficient. It aims to assess how well a monotonic function could be used to represent the relation between two variables. The correlation matrix describes the correlation and no correlation variance of a range of features through the values created in matrix ranging from +1 to −1. +1 means high and positively correlated, while −1 means less and negatively correlated. The features which are correlated are month and date, month and type of violation, latitude, and longitude. On the other hand, features highly less correlated are season and month. Additionally, the less correlated features also include season and date, season and type of violation, as shown in Figure 5. This indicates that month, latitudes, and longitude have a notable impact on the type of violation. The violations vary throughout the year as the month changes. Longitudes and latitudes show that the location of the violation also has a strong positive association with observed violations.

## 4. Conclusions

Traffic violations often caused by aggressive driving behavior are considered as significant indicators for crashes. Existing studies on aggressive driving behavior and violation analysis have mostly employed statistical regression and simulation-based techniques to explore factors responsible for such uncivilized driving behavior [40,44,45,46]. However, it is well-known that methods based on statistical analysis have a number of underlying assumptions and are unable to capture hidden correlation among explanatory variables [41,47]. Further, the low prediction accuracies obtained by these methods are also not highly reliable. Hence, in this study, we designed a systematic framework by first identifying violation hotspots using GIS, followed by classification and prediction of the different violations, using state-of-the-art ML methods. During the first phase of the study, a detailed descriptive analysis was conducted that showed that wrong-way driving had the highest proportion (75%) of total violations, whereas illegal parking had the lowest (2.10%). It was also noted that private cars and taxis were frequent violators. Similarly, temporal distribution analysis revealed that violations were more prevailing during the spring season, weekdays, and off-peak periods. The relationship between temporal attributes and occurrence of violation is consistent with a previous study [41]. Another recent study conducted by Liu et al. also indicated the relationship of time (peak hours/Off peak hours), month, and locations with a different type of violation occurrence [48]. Previous studies have also focused on the relationship between land-use and observed violations. During the second phase of the study, the Inverse Distance Weighted (IDW) method of interpolation in ArcMap GIS was used for the identification of hotspots for traffic violations along both expressways. Violation hotspots were mostly concentrated along commercial and public facility zone on S219, whereas, along S308 expressways, they were located mostly near the horizontal curve. Accurate identification of hotspots is vital for carrying subsequent treatment activities more efficiently within limited time and budget constraints. Studies suggest that the frequency of violations at a particular location may be associated with a range of factors, such as land-use, area type, time of the day, roadway design, weather conditions, and drivers’ socio-demographic attributes [40,49,50,51]. For example, another study, conducted by Zahid et al., suggested that risks of committing traffic violations are relatively more inside the central business districts (CBDs), dense residential landscape, the area with public facility services, and near urban intersections [41]. Lastly, during the third phase of the study, three different ML algorithms, i.e., KNN, SVM, and CN2 Rule Inducer, were applied for prediction and classification of traffic violations, considering spatio-temporal attributes of available data. The efficacy and predictive performance of proposed ML models were investigated using several classification evaluation metrics such as AUC, accuracy, precision, recall, F score, and specificity. Study results showed that KNN (k = 7) model using manhattan evualation had an accuracy of 99% outperformed SVM, and CN2 Rule Inducer. KNN model also showed increased predictive efficiency with reference to AUC, accuracy, precision, recall, F-score, and specificity. The outcome of this study could provide useful guidance to safety managers and practioners to initiate sound policy recommedations to enhance road safety.

The current study has a few limitations that must be` acknowledged and may be adressed in future studies. First, detailed demographic characteristics of drivers (such as gender, age, education, etc.) could be considered in future studies. Unfortunately, they were not available for this study. Second, the license plate record of the vehicles were not available in the current dataset, which limits its application to record the detailed history of violations comitted by individual vehicle/driver. This is a another major drawback that could provide valuable insights to in-depth violation analysis. Finally, it would be intresting to explore the impacts of operating styles, working hours, features of built environment, attributes of roadway geometric and daily driving distances on agressive driving behavior, and traffic violations in forthcoming studies.

## Figures and Tables

**Table 1:** K values for K Nearest Neighbors (KNN) model.

K (Number of Neighbors)	Metric	Weight
3	Euclidean	Distance
5	Euclidean	Distance
7	Manhattan	Distance
10	Euclidean	Uniform
12	Mahalanobis	Distance

**Table 2:** CN2 Rule Inducer setting parameters values.

Model Parameters	Parameter Value
α1	0.04
α2	0.04
Minimum rule coverage	1
Maximum rule coverage	7

**Table 3:** Descriptive Statistics of Violations (N = 2003).

Variable	Percentage of Total Violations (%)	Frequency	Variable Type
Wrongway driving	74.94	1501	Response
Violation of Prohibited Markings	17.87	358	Response
Overspeeding	4.54	91	Response
Illegal Parking	2.65	53	Response
**Vehicles Type**			
Private Car	58.46	1171	Predictor
Taxi	23.27	466	Predictor
Van	9.54	191	Predictor
Small Truck	6.19	124	Predictor
Bus	2.55	51	Predictor
**Seasons**			
Spring	54.72	1096	Predictor
Winter	34.4	689	Predictor
Summer	7.79	156	Predictor
Autumn	3.1	62	Predictor
**Week**			
Weekdays	68.3	1368	Predictor
Weekends	31.7	635	Predictor
**Hours of the Day**			
Peak Hours (9:00 a.m.–11:00 a.m., 15:00 p.m.–17:00 p.m.)	47.98	961	Predictor
Off Peak Hours (11:00 a.m.–15:00 p.m., 17:00 p.m.–9:00 a.m.)	52.02	1042	Predictor

## Appendix A

**Table A1:** Induced Rules.

No.	IF Condition	Then Class	Distribution	Probabilities (%)	Rule Quality	Length
1	Latitude ≥ 28.85656	Illegal parking	[12, 0, 0, 0]	81:6:6:6	0.929	1
2	Latitude ≤ 28.83151 and Season = Autumn	Illegal parking	[16, 0, 0, 0]	85:5:5:5	0.944	2
3	Latitude ≥ 28.83693 and Latitude ≤ 28.84196 and Season ≠ Summer	Illegal parking	[20, 0, 0, 0]	88:4:4:4	0.955	3
4	Latitude ≤ 28.83151 and Day of Week = Sunday	Illegal parking	[4, 0, 0, 0]	62:12:12:12	0.833	2
5	Lattitude ≤ 28.83151 and Day of Week = Tuesday	Illegal parking	[3, 2, 0, 0]	44:33:11:11	0.571	2
6	Lattitude ≤ 28.83151 and Season = Winter	Overspeeding	[0, 60, 0, 0]	2:95:2:2	0.984	2
7	Month ≥ 12.0 and Lattitude ≥ 28.84942	Overspeeding	[0, 22, 0, 0]	4:88:4:4	0.958	2
8	Lattitude ≥ 28.83693 and Minute ≥ 55.0	Overspeeding	[0, 3, 0, 0]	14:57:14:14	0.8	2
9	Day of Week = Sunday and Hour ≥ 14.0	Violation of prohibited markings	[0, 0, 76, 5]	1:1:91:7	0.928	3
10	Month ≤ 2.0 and Vehicle type = Taxi/Passenger Car and Day of Week = Monday	Violation of prohibited markings	[0, 0, 35, 12]	2:2:71:25	0.735	3
11	Month ≤ 2.0 and Day of Week = Monday	Violation of prohibited markings	[0, 0, 100, 79]	1:1:55:44	0.558	2
12	Month ≤ 2.0 and Day of Week = Sunday	Violation of prohibited markings	[0, 0, 35, 36]	1:1:48:49	0.493	2
13	Month ≤ 2.0 and Day of Week = Tuesday	Violation of prohibited markings	[0, 0, 16, 23]	2:2:40:56	0.415	2
14	Season = Autumn and Month ≥ 10.0	Violation of prohibited markings	[0, 0, 4, 0]	12:12:62:1	0.833	3
15	Hour ≥ 17.0 and Season = Spring	Wrongway driving	[0, 0, 2, 151]	1:1:2:97	0.981	2
16	Day of Week = Wednesday and Season = Spring	Wrongway driving	[0, 0, 4, 113]	1:1:4:94	0.958	2
17	Day of Week = Saturday and Peak/Off Peak ≠ Peak	Wrongway driving	[1, 0, 7, 121]	2:1:6:92	0.931	2
18	Season ≠ Winter and Day of Week = Friday	Wrongway driving	[3, 0, 6, 141]	3:1:5:92	0.934	2
19	Day of Week = Thursday and Season = Spring	Wrongway driving	[0, 0, 7, 104]	1:1:7:91	0.929	2
20	Day of Week = Sunday and Hour ≥ 10.0	Wrongway driving	[0, 0, 2, 105]	1:1:3:95	0.972	3
21	Season ≠ Winter	Wrongway driving	[39, 9, 70, 209]	12:3:21:63	0.638	1
22	Month ≤ 2.0 and Day of Week = Thursday	Wrongway driving	[0, 0, 1, 24]	3:3:7:86	0.926	2
23	Month ≤ 2.0 and Month ≥ 2.0	Wrongway driving	[0, 0, 16, 107]	1:1:13:85	0.864	2
24	Month ≤ 5.0 and Day of Week = Friday	Wrongway driving	[2, 0, 21, 29]	5:2:39:54	0.556	2
25	Month ≤ 5.0 and Day of Week = Tuesday	Wrongway driving	[0, 0, 23, 23]	2:2:48:48	0.5	2
26	Day_of_Week = Monday and Hour ≤ 11.0	Wrongway driving	[2, 2, 19, 25]	6:6:38:50	0.52	2
27	Month ≤ 5.0 and Day of Week = Saturday	Wrongway driving	[0, 0, 15, 12]	3:3:52:42	0.448	2
28	Month ≤ 2.0 and Hour ≥ 17.0	Wrongway driving	[0, 0, 13, 10]	4:4:52:41	0.44	2
29	Day of Week = Wednesday	Wrongway driving	[8, 3, 10, 12]	24:11:30:135	0.371	1
30	TRUE	Wrongway driving	[57, 91, 358, 1502]	3:5:18:75	0.747	
